# Role of N-Nitro-L-Arginine-Methylester as anti-oxidant in transient cerebral ischemia and reperfusion in rats

**DOI:** 10.1186/2040-7378-5-1

**Published:** 2013-01-04

**Authors:** Hiba A Awooda, Mohamed F Lutfi, Gihan M Sharara, Amal M Saeed

**Affiliations:** 1 Department of Physiology – Faculty of Medicine and Heath Sciences, Alneelain University, Khartoum, Sudan; 2 Department of Biochemistry– Faculty of Medicine and Heath Sciences, Alexandria University, Alexandria, Egypt; 3 Department of Physiology – Faculty of Medicine and Heath Sciences, University of Khartoum, Khartoum, Sudan

**Keywords:** Cerebral, Ischemia/reperfusion, L-NAME, Malondialdehyde, Nitric oxide, Total antioxidant capacity

## Abstract

**Background:**

Previous reports assessing the neuroprotective role of nonselective Nitric Oxide synthase (NOS) inhibitor N-nitro-L-arginine-methylester (L-NAME) following cerebral ischemia/reperfusion are contradictory. The aim of this work was to examine the potential benefits of L-NAME on rats subjected to transient focal cerebral ischemia/reperfusion.

**Methods:**

The study involved 30 adult male Wistar rats divided into three groups 10 rats in each: First group was sham-operated and served as a control, a ischemia/reperfusion (I/R) group of rats infused with 0.9% normal saline intraperitoneally 15 minutes prior to 30 minutes of left common carotid artery (CCA) occlusion and a test group infused with L-NAME intraperitoneally 15 minutes prior to ischemia. Neurobehavioral assessments were evaluated and quantitative assessment of malondialdehyde (MDA), Nitric oxide (NO) metabolites and total antioxidant capacity (TAC) in both serum and the affected cerebral hemisphere were achieved.

**Results:**

Rats’ neurological deficit and TAC were significantly decreased while NO and MDA were significantly increased in the I/R compared with the control group (P < 0.001). Alternatively in the L-NAME group, neurological deficit and TAC were significantly improved while NO and MDA were significantly decreased compared to I/R group (P < 0.001).

**Conclusions:**

L-NAME pretreatment for rats undergoing cerebral ischemia/reperfusion significantly improves neurological deficit while reducing oxidative stress biomarkers in the affected cerebral hemisphere.

## Introduction

NO is synthesized from its precursor L-arginine by the action of NOS and proved to be a vital cellular signaling molecule in the central nervous system [1]. Three NOS isoforms have been identified in the brain following the onset of cerebral ischemia [2]; however, their exact pathophysiological role in cerebral ischemia/reperfusion is uncertain [3,4]. NO produced by endothelial NOS (eNOS) was proved to be beneficial during cerebral ischemia/reperfusion. This is because of its vasodilator and antiplatelet effects [5]. In addition, NO increases vascular smooth muscle proliferation and migration, so enhances angiogenesis after stroke [6]. In contrast, the neuronal and inducible isoforms of NOS (nNOS, iNOS) can be neurotoxic [7], probably because they enhance peroxynitrite production, a free radical involved in lipid peroxidation, suppress mitochondrial respiratory enzymes and damage DNA. NO can directly inhibit enzymes needed for mitochondrial respiration, glycolysis and DNA replication [8]. Accordingly, inhibition of NOS and hence decreasing NO production may be valuable while treating patients with acute stroke. However, previous studies evaluating the possible effects of nonselective NOS inhibitor L-NAME in the experimental stroke model are contradictory. The results ranged from reduction [9] to no effect [10] or even a worsening of ischemic neuronal damage [11,12]. This study aimed to explore the potential anti-oxidant effect of L-NAME in rats with transient focal cerebral ischemia/reperfusion.

## Materials and methods

### Animals

The animals were handled in accordance with the ethical standards laid down in the US National Institutes of Health (NIH Publication No. 85–23) and its later revisions. Male Wistar rats, weighing 150–250 g were selected and preserved at a constant temperature of 22±2°C with a fixed 12:12-h light–dark cycle. Nutritionally balanced pellets and water were freely available.

### Experimental design

Three randomly divided experimental groups were used, of 10 rats each: (1) sham operated: which included full surgical preparation without common carotid artery (CCA) occlusion serve as control group (2) ischemia/reperfusion (I/R) group: Brain ischemia was maintained for 30 minutes of left CCA occlusion followed by 24 hours reperfusion. All rats in this group received 0.9% normal saline intraperitoneally 15 minutes prior to induction of ischemia [11]. The volume of saline infused was equivalent to the volume of L-NAME received by the third group.

(3) L-NAME (Sigma aldrich Co., Ltd.) 15 mg/kg over 5 minutes intraperitoneally (i.p) 15 minutes before left CCA occlusion [11].

### Cerebral ischemia induction

The animals were fasted overnight prior to surgery with free access to tap water. Anesthesia was induced by ether inhalation and maintained by thiopental sodium (2.5 mg/kg) [13]. Body temperature was kept constant at 36.5±0.5°C using heating pad. A longitudinal cervical incision (2 cm) was made lateral to the midline and the CCA was carefully dissected. Ischemia was induced by placing non traumatic microvascular clip on left CCA just prior to its bifurcation [14]. During ischemia rats were monitored for body temperature and respiration pattern. The vascular occlusion was maintained for 30 minutes, and then the clips were removed to resume blood flow to the ischemic region for 24 hours [11]. Finally, the incisions were sutured, the animal was allowed to recover from anesthesia, and returned to a warm cage for recuperation during reperfusion period.

### Neurological and behavioral evaluation

Neurobehavioral tests of all experimental groups were assessed daily to determine the effect of ischemic injury on them. Neurobehavioral evaluations were performed three times: the day before surgery, the same day as surgery and just before killing the animals. Each rat was examined in the late afternoon hours so that rats that had been operated in the morning would fully recovered from the effects of anesthesia by the time of evaluation. The neurobehavioral study consisted of the following six tests: spontaneous activity, symmetry in the movement of the four limbs, forepaw outstretching, climbing, body proprioception and response to vibrissae touch. The score given to each rat at the end of the evaluation is the summation of all six individual test scores. The minimum neurological score was 3 and the maximum was 18 [15].

### Laboratory investigations

At the end of experimental period, the rats were sacrificed by decapitation. Brains were rapidly removed from the skull and washed with cold saline and stored at −20°C for further analysis. A small part of each brain from the affected hemisphere was dissected to approximately 1–2 mm pieces and they were homogenized in 7 ml of ice-cold extraction buffer (1% Triton X-100, 10 mmol/l MgSO4, 1 mmol/l EDTA, 1 mmol/l dithiothreitol, 0.5 mol/l NaCl,1% protease inhibitor cocktail, 20 mmol/l HEPES and pH of 7.5) [16]. The homogenate was centrifuged; the supernatant was taken and stored at −20°C until being used. A modification of the method of Lowry was used for the determination of protein in the brain homogenate [17]. The level of the NO metabolites (nitrite and nitrate) [18,19] and total antioxidant capacity (TAC) were measured colorimetrically. Satoh method was used to measure serum and brain homogenate malondialdehyde (MDA) levels (an indicator of lipid peroxidation) [20].

### Data analysis

Statistical evaluation was performed using the Microsoft Office Excel (Microsoft Office Excel for windows; 2003) and SPSS (SPSS for windows version 19). Screening studied rats’ groups for significant difference in the mean of MDA, NO and TAC was performed using analysis of variance. P < 0.05 was considered significant.

## Results

**Figure 1:**
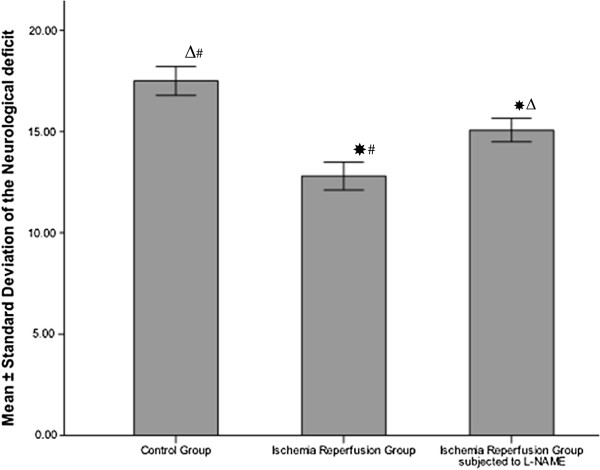
**Neurological deficit of sham operated and in ischemia/reperfusion rats with and without L-NAME.** * Significant with controls, ^Δ^ Significant with ischemia reperfusion, ^#^ Significant with L-NAME.

**Figure 2:**
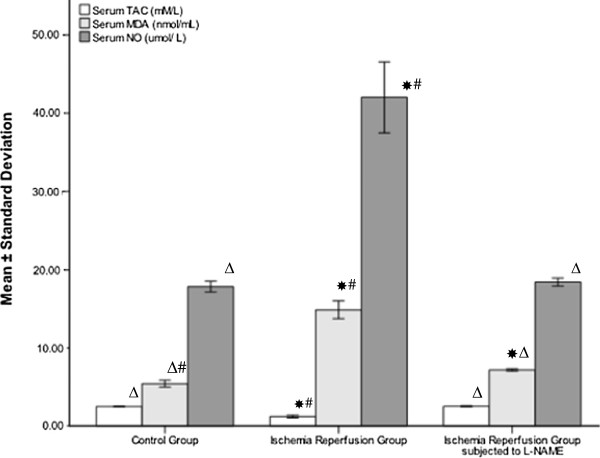
**Serum level of MDA, NO and TAC in sham operated and in ischemia reperfusion rats with and without L-NAME.** * Significant with controls, ^Δ^ Significant with ischemia reperfusion, ^#^ Significant with L-NAME.

As shown in Figure 1, the means of the neurological scores of both I/R (12.798±0.689) and L-NAME (15.07±0.584) were significantly lower compared to the control group (17.50±0.707, *P* < 0.001). The L-NAME group showed a significant improvement in neurological deficit compared to the I/R group (*P* < 0.001). In addition, I/R group demonstrated a significant increase in the serum levels of both MDA and NO (14.88±1.14 nmol/mL, 42.03±4.558 μmol/L respectively) compared to the control group (5.43±0.44 nmol/mL, 17.84±0.701 μmol/L respectively, *P* < 0.001). Alternatively, the L-NAME group showed significant decrease in serum level of MDA and NO (7.18±0.135, 18.44±0.513 μmol/L respectively, *P* < 0.001) compared to the I/R group (Figure 2). The serum level of TAC in the I/R group (1.21±0.169 mM/L) was significantly lower compared to the control group (2.52±0.062 mM/L, *P* < 0.001) but not L-NAME group (2.53±0.067 mM/L, P = 0.858) (Figure 2). L-NAME pretreatment resulted in significant higher serum level of TAC compare to rats treated with normal saline (*P* < 0.001).

**Figure 3:**
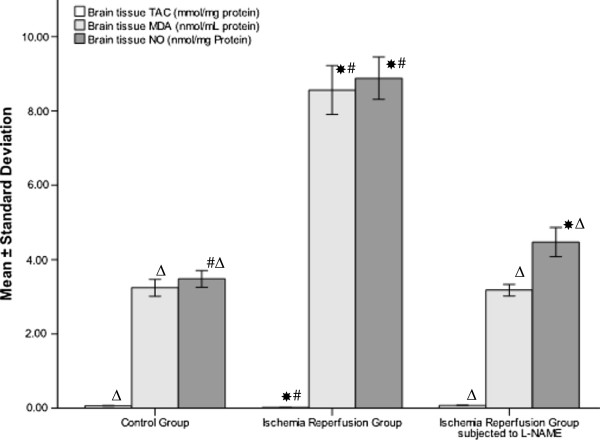
**Brain tissue level of MDA, NO and TAC in sham operated and in ischemia reperfusion rats with and without L-NAME.** * Significant with controls, ^Δ^ Significant with ischemia reperfusion, ^#^ Significant with L-NAME.

Regarding brain tissue levels of MDA and NO, the I/R group demonstrated a significant increase in the tissue level of both MDA and NO (8.56±0.658, 8.88±0.572 nmol/mg protein) compared to the control group (3.24±0.226, 3.48±0.228 nmol/mg protein, *P* < 0.001). The L-NAME group showed a significant decrease in tissue level of MDA and NO (3.18±0.155, 4.47±0.392 nmol/mg protein, *P* < 0.001) compared to the I/R group. The brain TAC level of the I/R group (0.0186±0.00373 mmol/mg protein) was significantly decreased compared to the control group (0.070±0.0085 mmol/mg protein, *P* < 0.001) but not the L-NAME group (0.0747±0.00563 mmol/mg protein, *P* = 0.112). On the contrary, administration of L-NAME prior to ischemia result in significant increase of brain TAC level compare to rats subjected to saline infusion (*P* < 0.001) (Figure 3).

## Discussion

It is evident from the current findings that oxidative stress and inflammation are predictable outcome following cerebral ischemia/reperfusion. The biomarkers of the offending pathological mechanisms involved in oxidative stress and neuroinflammation were significantly lowered by L-NAME administration prior to cerebral ischemia/reperfusion. This fact suggested a probable anti-oxidant effect of L-NAME which was evident by the improvement of the neurological score of rats treated by this medication prior to cerebral ischemia/reperfusion.

Previous studies carried out to evaluate the effect of L-NAME in rats subjected to focal or global cerebral ischemia showed no agreement [21,22], probably due to the differences between study protocols in terms of animal species, physiological parameters measured, surgical approach, duration of ischemia and dosing regimen [9,22].

Considerable researches were done to determine the best dosing and timing regimen of L-NAME in terms of neuroprotection [22-24]. Based on previous reports, the current study used probably the best dosing and timing regimen of L-NAME. The role of NO in cerebral ischemia was examined 24 hours after CCA occlusion using a dose of 15 mg/kg L-NAME, 15 minutes prior to induction of cerebral ischemia. Ding-Zhou *et al*. and others, tried different doses of L-NAME and they found that low doses (1, 3 and 10 mg/kg) reduced the infarct volume, whereas a high dose (30 mg/kg) killed 80% of the studied animals. [9,25,26]. On other hand, Batteur-Parmentier *et al*. proposed that eNOS contribution to the early NO production after ischemia may have a beneficial effect by increasing cerebral blood flow, decreasing platelet aggregation and neutrophils adhesion [23]. However, L-NAME given during the acute phase of transient ischemia might fail to reduce ischemic infarct and even aggravated the ischemic outcome in models of cerebral ischemia [25,26].

The results of the current study are further supported by the systematic review conducted by Willmot and his group [27] which analyzed 2321 models of cerebral ischemia and proved the beneficial effects of NOS inhibitors including L-NAME. These beneficial effects include the reduction of the cerebral lesion size and improved neurological deficit due to reduced formation of peroxynitrite and reactive oxygen species (ROS) [28] inhibition of brain edema, reduced vascular damage, inhibition of apoptosis and necrosis [9]. It is worth mentioning that inhibition of NO production induces vasoconstriction and subsequently increases blood pressure. However, Ashwal *et al*. demonstrated that low dose of L-NAME had neuroprotective outcome without influencing the blood pressure [29]. These suggestions were compatible with Mohammadi *et al*. report that neuroprotective potential of a low dose of L-NAME as indicated by improving the neurological deficit, reducing cerebral edema and the infarction volume, decreased cerebral vascular permeability, with no significant change in the value of mean arterial blood pressures or regional cerebral blood flow [12].

Other researches with comparable findings to the current study regarding the antioxidant effect of L-NAME on oxidative stress biomarkers include Seif-el-Nasr [30] and Sayan *et al*. [31]. Seif-el-Nasr assessed the antioxidant effect L-NAME in a model of global cerebral ischemia induced by 60 min of bilateral ligation of the CCA followed by 60 min of reperfusion period [30]. L-NAME (1 and 3 mg/kg, twice after ischemia and 15 min before termination of the experiment) produced a significant decrease in level of lipid peroxidation in the rats’ brains. Sayan *et al*. proposed that L-NAME significantly reduced MDA in rat’s model of sciatic nerve ischemia/reperfusion [31]. In contrast, Adaramoye *et al*. reported that as consequence of L-NAME treatment (40 mg/kg 5 times in a week for a period of 3 weeks), various oxidative stress parameters in the blood, kidney, liver and heart were enhanced [32]. Similarly, L-NAME provoked oxidative stress in rat’s model of hind legs ischemia/reperfusion [33].

In conclusion, the current study demonstrates the potential anti-oxidant effect of L-NAME in rat’s focal cerebral ischemia/reperfusion, through inhibition of oxidative stress and lipid peroxidation as well as improvement of neurological deficits.

## Competing interest

On behalf of all authors, the corresponding author states that there is no conflict of interest.

## Authors’ contributions

HAA conceived, designed the study and carried out all the experimental work and prepared the manuscript. GMS carried out all the Laboratory investigations. MFL and AMS carried out statistical analysis and helped to draft the manuscript. All authors read and approved the final manuscript.
